# Meary angle for the prediction of mitral valve prolapse risk in non-syndromic patients with pes planus, a cross-sectional study

**DOI:** 10.1186/s13104-022-06032-0

**Published:** 2022-04-25

**Authors:** Antoine AbdelMassih, Rafeef Hozaien, Fady Mishriky, Mark Michael, Mostafa AmanAllah, Nada Ali, Nadine ElGamal, Omar Medhat, Mona Kamel, Rasha Helmy, Mai Sarhan, Hams Attalla, Omneya Dawoud, Athar Marwan, Mohamed Ghobashy

**Affiliations:** 1 Pediatrics’ Department, Faculty of Medicine, Cairo University, Giza, Egypt; 2 Research Accessibility Team: Students’ and Interns’ Research Program, Faculty of Medicine, Cairo University, Giza, Egypt; 3 Family Medicine Department, Faculty of Medicine, Cairo University, Giza, Egypt; 4 Pediatrics’ Department, Faculty of Medicine, Helwan University, Helwan, Egypt; 5 Radiology Department, Faculty of Medicine, Cairo University, Giza, Egypt; 6 MD training program, Faculty of medicine, Cairo University, Cairo, Egypt

**Keywords:** MVP, Pes planus, Meary angle

## Abstract

**Objectives:**

Mitral Valve Prolapse (MVP) is a common valvular abnormality accounting for 2% of the population. There is a reported association between pes planus (PP) and MVP in some syndromes such as Marfan. However, this association has not been tested in non-syndromic cases. The primary outcome of this study is to measure the prevalence of MVP in a population of patients with PP. The secondary outcome parameter is to determine if the Meary angle (MA), a measure of the severity of flat foot, can be effectively used in the prediction of the presence of MVP. Forty-one patients with PP were screened using a lateral x-ray foot to determine MA while echocardiography was utilized to identify the presence and grade of MVP.

**Results:**

88% of screened patients were diagnosed with MVP. MA was correlated with the grade of MVP and showed high diagnostic accuracy (sensitivity 100% and specificity 90%) in predicting MVP risk when higher than 5. Children with PP are at a higher risk for MVP than the general population. Accordingly, the utilization of MA in such a specific population for the determination of patients at a higher need for echocardiography seems to be a worthwhile strategy in diagnosing MVP.

**Supplementary Information:**

The online version contains supplementary material available at 10.1186/s13104-022-06032-0.

## Introduction

Mitral valve prolapse (MVP) is a myxomatous degeneration of the mitral valve characterized by the displacement of the abnormally thickened mitral valve leaflet into the left atrium during systole. Though generally sporadic, MVP is also associated with a variety of congenital disorders of the connective tissue, including Marfan syndrome (MS), Ehler-Danlos, syndrome, osteogenesis imperfecta, dominant cutis laxa pseudoxanthoma elasticum, and MASS syndrome (mitral valve prolapse, aortic root dilatation, skeletal changes, and skin changes) [1].

Pes planus or flat foot is a hallmark of connective tissue disorders. When screened by De Maio and colleagues, the skeletal abnormalities associated with Marfan syndrome were found to be in 21% of the study subjects. Moreover, defects in fibrillin or collagen genes have been implicated in the development of flat foot as well as mitral valve prolapse. Normally, the angle between the midline axis of the talus with the midline axis of the first metatarsal is in line at 0º . This angle is termed Meary’s angle (MA). MA is used for the grading of flat foot, where an angle greater than 0 but less than 15 degrees signifies a mild deformity; an angle ranging from 16 to 30 is considered moderate, while an angle exceeding 30 denotes severe PP. The measurement of MA requires a simple and affordable lateral x-ray foot [2].

Despite the established etiology shared between PP and MVP, to our knowledge, no study to date has explored the prevalence of MVP in a population of patients with non-syndromic PP.

Moreover, no study has explored the potential use of MA in the prediction of MVP in patients with flat foot [3].

The primary outcome parameter of this study is to determine the prevalence of MVP in patients with PP. The secondary outcome parameters include the determination of the cutoff level of MA to predict MVP.

## Main text

### Methods

#### Study subjects

This study was designed as a cross-sectional study. All patients attending the outpatient clinic of Cairo University Children Hospital fulfilling the definition of PP [4] (the absence of the medial longitudinal arch during weight-bearing) were included in the study. The exclusion criteria comprised of arrhythmia, congenital or acquired heart disease, or any concurrent systemic and connective tissue disease (obesity, diabetes, and systemic lupus erythematosus, MS). Furthermore, any child with more than 3 dysmorphic features was excluded.

#### Study methods

-History taking including age and sex.
*-Anthropometric measurements such as weight:* Weight was measured without clothing or diaper to 0.5-g accuracy on a balance scale (MARSDEN, Rotherham, UK) [5].
-Radiographic assessment of MA.

Meary’s angle (MA) or talus-first metatarsal angle has been used to identify the severity of PP on lateral weight-bearing foot radiographs. It is the angle between a line drawn along the longitudinal axes of the talus (mid-talar axis) and the first metatarsal (first metatarsal axis) [6]. (The method of measurement has been described in Additional file 1: Figure S1).

In the normal weight-bearing foot, the midline axis of the talus is in line with the midline axis of the first metatarsal. Normally the Meary’s angle is 0.

The severity of PP was classified as follows [3, 6]:

-Mild: < 15º.
-Moderate: 16–30º.
-Severe: > 30º.
-Echocardiographic grading of mitral valve prolapse.
- Cohen et al. grading of mitral valve prolapse has been employed using the Cohen et al. method as follows [7]:
-Grade I or “mild” prolapse = the maximally prolapsed segment of one or both mitral leaflets or their point of coaptation extended is 5 mm or less above the plane of the mitral ring in either the parasternal long axis (PSLA) and/or apical four-chamber view. One or both leaflets moved sufficiently above the ring plane to create an echo-free space between the ring plane and the leading edge of the leaflet echoes.
-Grade II or “moderate” prolapse = the maximally prolapsed segment of one or both mitral leaflets or their point of coaptation extended is > 5 mm above the plane of the mitral ring in either the PSLA and/or apical four-chamber view.
-Grade III or “severe” prolapse = prolapse meeting grade II criteria; in addition, one or both leaflets showed evidence of redundancy defined as the infolding of tissue on itself in any of PSLA or apical view.

### Statistical analysis

Data was analyzed using IBM# SPSS# Statistics version 23 (IBM# Corp, Armonk, NY, USA). Data was tested for normality using the Kolmogorov–Smirnov test. Furthermore, data was normally distributed and presented as mean and standard deviation, while categorical variables were expressed as numbers and percentages (Table 1).

**Table 1:** Characteristics of the study subjects

Sex n/%	Male n = 22, 53.7% Female n = 19, 46.3%
Age mean ± SD	8.2 ± 0.9
Weight in kg mean ± SD	24 ± 5
Height	
MVP prevalence n/%	88% n = 32
MVP grades n/%	No MVP n = 9, 22% MVP grade I n = 11, 27% MVP grade II n = 16, 39% MVP grade III n = 5, 12%
MA (mean ± SD)	9 ± 4

*cm* centimeter, *MA* meary angle, *MVP* mitral valve prolapse, *kg* kilogram, *n* number, % percentage

**Fig. 1:**
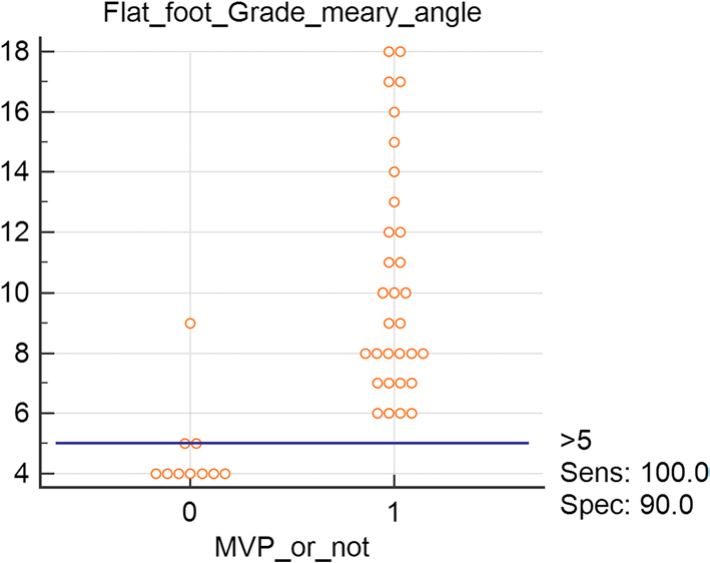
Interactive dot diagram to show the diagnostic accuracy of Meary angle in predicting MVP. *Y axis* Meary angle, X axis: *MVP* mitral valve prolapse (0: absent/1: present). *Sens* sensitivity, *Spec* specificity

Receiver-operating characteristic (ROC) curve analysis was performed and illustrated as an interactive dot diagram to show the diagnostic accuracy of MA in predicting the risk of MVP in children with flat foot (Fig. 1).

P-Value < 0.05 was considered statistically significant.

## Results

Table 1 shows the characteristics of the study subjects. A high prevalence of MVP was found in patients with PP (88%).

Figure 1 is an interactive dot diagram, to illustrate the ROC analysis performed to test the diagnostic accuracy of meary angle in predicting the presence or absence of MVP.

Given this tight relationship, we performed a ROC analysis to determine the sensitivity and specificity of MA in determining the risk of MVP. MA achieved a high sensitivity of 100%, and specificity in predicting the risk of MVP as shown in the interactive dot diagram of Fig. 1. The negative predictive value of an angle > 5 in excluding MVP was 100%, while the positive predictive value was 52% in predicting the presence of MVP.

## Discussion

The association between mitral valve prolapse and skeletal abnormalities has been the focus of several previous studies, almost all of which have concentrated on back or thoracic skeletal abnormalities. Notably, Movahed and colleagues reported an association between kyphosis and MVP. Indeed, straight back was coexistent in 27% of patients with myxomatous mitral valve degeneration [8].

Several former studies by Salomon et al., and Peh et al. have reported an association between thoracic skeletal abnormalities such as pectus excavatum and mitral valve prolapse. To date, the etiology of the close association between skeletal abnormalities and MVP has not been investigated. Although it has been postulated that this association might constitute an attenuated form of Marfan syndrome, this speculation remains elusive in the absence of any genetic study to prove it [9, 10].

To our knowledge, no study to date has investigated the association between pes planus and MVP, despite the fact that they might be caused by similar connective tissue dysfunction.

Our study notes an unexpectedly high prevalence of MVP in patients with flat foot (88%). This association suggests a common genetic background between both anomalies. This coincides with Raj and Kiel’s report about the prevalence of PP and its genetic background, where MVP prevalence in the general population ranges from 1 to 37% [4]. The mean age of our patients was 8.2 ± 0.9, which excludes the entity of transient pes planus that usually disappears before the age of 6 years [4].

There is a reported 2.5% risk of sudden death with mitral valve prolapse, mostly due to associated Long QT syndrome. Furthermore, there are speculations that the tension on the chordae and subvalvular apparatus caused by the prolapsing valve results in myocardial degeneration and fibrosis, with the subsequent creation of a substrate for arrhythmias. The mentioned risk has prompted a remarkable need for the development of a screening policy for MVP. Three-generational screening of families in the case of a diagnosed family member with MVP has been recently proposed, but the cost-effectiveness of this strategy remains elusive [11, 12].

Alternatively, our study offers a new screening policy for MVP, where we suggest the determination of MA in patients with PP. MA angle in lateral foot xray is a routine procedure in such types of patients and will not add a non-required investigation among them. An angle greater than 5 degrees, according to our findings, has a 100% sensitivity in pointing to the concurrent presence of MVP, which is a justified diagnostic accuracy for performing echocardiography in this high-risk group.

To conclude, the tight relationship between pes planus and MVP illustrated in this study warrants the screening of patients with pes planus for the presence of MVP. Meary angle, an easy and cheap radiographic measure, can serve as a good predictor of MVP. Nonetheless, the generalization of the results of this study needs a larger sample size to consolidate the revealed results.

## Limitations

The main limitation to the generalization of the results of this study is the relatively small sample size. Therefore, larger sample sizes are needed to consolidate the results.

Another limitation was the absence of genetic screening for fibrillin mutations in the study subjects. Correspondingly, we are planning to study the genetic background of the association between pes-planus and mitral valve prolapse away from the classic Marfan-causing mutations.

According to Jon’s method of assessment of Meary angle [6], a minimum age of 5 years was required for assessment of such angle, this means that relying on Meary angle might delay screening of MVP until this age.

## Supplementary Information

**Additional file 1:** **Figure S1.** Title: plain XRAY showing Meary angle 13 degrees.

## Data Availability

Available upon request, the corresponding author of the article Antoine AbdelMassih can supply it (email: antoine.abdelmassih@kasralainy.edu.eg).

## Abbreviations

IBM: International business machines corporation
MA: Meary angle
MASS: Mitral valve prolapse, aortic root dilatation, skeletal changes, and skin changes
MS: Marfan syndrome
MVP: Mitral valve proplapse
NY: New York
PP: Pes planus
PSLA: Parasternal long axis
ROC: Receiver operating characteristics
SPSS: Statistical package for the social sciences
UK: United Kingdom
