# Supplementary figures and images for: Meary angle for the prediction of mitral valve prolapse risk in non-syndromic patients with pes planus, a cross-sectional study
Source: BMC Res Notes. 2022 Apr 25;15:145. doi: 10.1186/s13104-022-06032-0 (PMC9036702; doi:10.1186/s13104-022-06032-0)

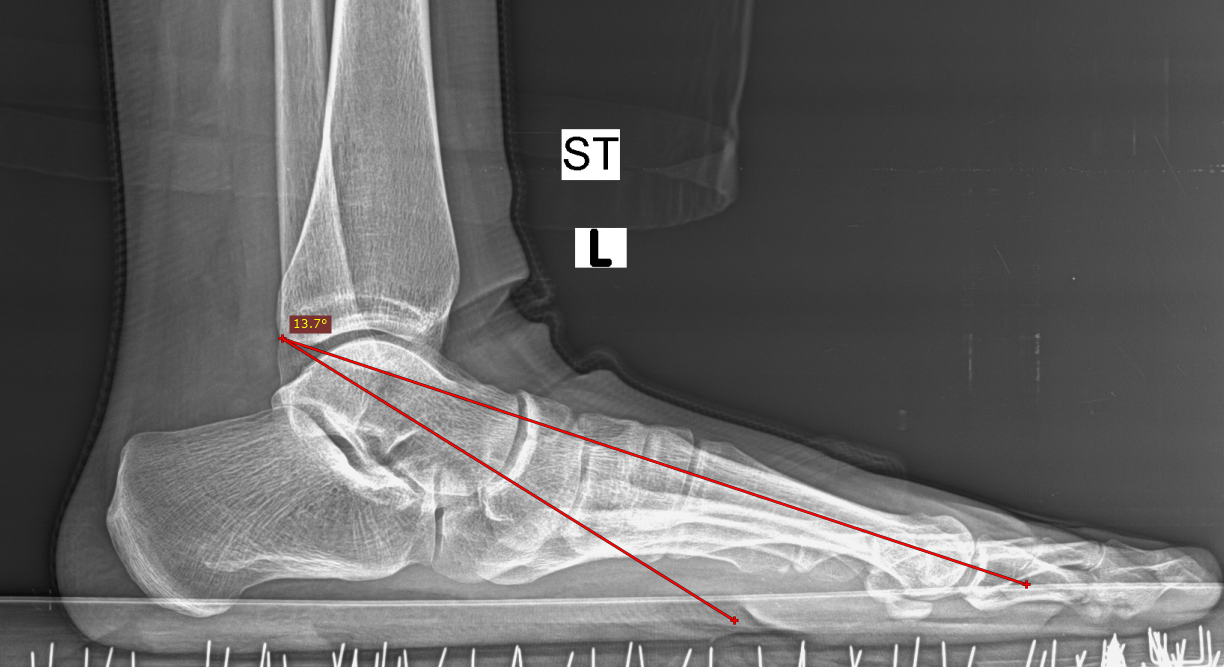

Supplement: Supplementary file 1 — Additional file 1: Figure S1. Title: plain XRAY showing Meary angle 13 degrees. [file 13104_2022_6032_MOESM1_ESM.tif]
